# The size distribution and origin of elements bound to ambient particles: a case study of a Polish urban area

**DOI:** 10.1007/s10661-015-4450-5

**Published:** 2015-04-11

**Authors:** Wioletta Rogula-Kozłowska, Grzegorz Majewski, Piotr Oskar Czechowski

**Affiliations:** Institute of Environmental Engineering, Polish Academy of Sciences, 34 M. Skłodowska-Curie St., 41-819 Zabrze, Poland; Division of Meteorology and Climatology, Warsaw University of Life Sciences, 166 Nowoursynowska St., 02-776 Warszawa, Poland; Information Systems Department, Gdynia Maritime University, 83, Morska St., 81-225 Gdynia, Poland

**Keywords:** Ultrafine ambient aerosol, Particulate matter, Toxic metals, Mass size distribution, PCA, Cluster analysis

## Abstract

Ambient particulate matter (PM) was sampled in Zabrze (southern Poland) in the heating period of 2009. It was investigated for distribution of its mass and of the masses of its 18 component elements (S, Cl, K, Ca, Cr, Mn, Fe, Ni, Cu, Zn, Ge, As, Br, Sr, Cd, Sb, Ba, and Pb) among 13 PM size fractions. In the paper, the distribution modality of and the correlations between the ambient concentrations of these elements are discussed and interpreted in terms of the source apportionment of PM emissions. By weight, S, Cl, K, Ca, Cr, Mn, Fe, Ni, Cu, Zn, Ge, As, Br, Sr, Cd, Sb, Ba, and Pb were 10 % of coarse and 9 % of ultrafine particles. The collective mass of these elements was no more than 3.5 % of the mass of the particles with the aerodynamic diameter *D*_p_ between 0.4 and 1.0 μm (PM_0.4–1_), whose ambient mass concentration was the highest. The PM mass size distribution for the sampling period is bimodal; it has the accumulation and coarse modes. The coarse particles were probably of the mineral/soil origin (characteristic elements: Ca, Fe, Sr, and Ba), being re-suspended polluted soil or road dust (characteristic elements: Ca, Fe, Sr, Ba, S, K, Cr, Cu, Zn, Br, Sb, Pb). The maxima of the density functions (modes) of the concentration distributions with respect to particle size of PM-bound S, Cl, K, Cu, Zn, Ge, Br, Cd, Sb, and Pb within the *D*_p_ interval from 0.108 to 1.6 μm (accumulation PM particles) indicate the emissions from furnaces and road traffic. The distributions of PM-bound As, Mn, Ba, and Sr concentrations have their modes within *D*_p_ ≤ 0.108 μm (nucleation PM particles), indicating the emissions from high-temperature processes (industrial sources or car engines). In this work, principal component analysis (PCA) is applied separately to each of the 13 fraction-related sets of the concentrations of the 18 PM-bound elements, and further, the fractions are grouped by their origin using cluster analysis (CA) applied to the 13 fraction-related first principal components (PC1). Four distinct groups of the PM fractions are identified: (PM_1.6–2.5_, PM_2.5–4.4_,), (PM_0.03–0.06_, PM_0.108–0.17_), (PM_0.06–0.108_, PM_0.17–0.26_, PM_0.26–0.4_, PM_0.4–0.65_, PM_0.65–1_, PM_1–1.6_), and (PM_4.4–6.8_, PM_6.8–10_, PM_>10_). The PM sources attributed to these groups by using PCA followed by CA are roughly the same as the sources from the apportionment done by analyzing the modality of the mass size distributions.

## Introduction

Airborne particles can absorb practically any air contaminant. This capability of particulate matter (PM), due to its physicochemical properties, makes PM a great threat to human health, especially in big cities (Molina and Molina 2004; Lee and Lee 2008; Kong et al. 2012). The dependence between the ambient concentrations of fine PM (PM_1_ or PM_2.5_, particles with the aerodynamic diameter *D*_p_ ≤ 1 or 2.5 μm, respectively) and the PM health effects is well-studied and obvious: elevation of the PM concentrations increases mortality and morbidity in exposed populations (Pope et al. 2002; López-Villarrubia et al. 2012). However, the exact mechanisms of the PM toxic action on living organisms remain unknown (Dreher 2000; Harrison and Yin 2000). The PM toxicity derives rather from the synergistic effects of physicochemical properties of the particles and the environmental circumstances (Saldiva et al. 2002; Wellenius et al. 2003); nevertheless, the elemental content of PM is proved to decide on the potential health effects of PM.

The hazard from the PM-bound, so called, toxic or heavy metals is partially due to the chemical forms of their occurrence (Costa and Dreher 1997; Na and Cocker 2009; Daher et al. 2012; Rogula-Kozłowska et al. 2013a). In general, being easily water-soluble, they become bioavailable after entering water or soil.

Table 1 presents the elemental composition of various PM fractions at a number of sites in Europe. The PM component concentrations are averaged over various periods of the last decade, and come from urban or urban background sites.

**Table 1:** Elemental compositions of various fractions of PM in urban locations (urban background/city center) in Europe during the last 10 years

Data source	City (country)	Averaging period	PM fraction	Average concentration, ng/m^3^												
				PM^a^	V	Cr	Mn	Fe	Co	Ni	Cu	Zn	As	Cd	Ba	Pb
(Aldabe et al. 2011)	Navarra (Spain)	I–XII 2008	PM_10_	25.91	–	2.81	6.88	–	1.20	2.21	26.79	29.25	0.21	0.04	18.91	3.33
(Aldabe et al. 2011)	Navarra (Spain)	I–XII 2008	PM_2.5_	15.38	–	2.39	2.57	–	0.99	1.31	11.98	17.98	0.16	0.05	12.08	2.29
(Minguillón et al. 2012)	Barcelona (Spain)	XI 2008–XI 2009	PM_2.5_	20	12.7	3.4	38.0	400	0.2	7.1	45.9	61.0	2.0	0.4	5.2	10.2
(Chen et al. 2013)	Los Barrios (Spain)	2009	PM_10_	n.m.	–	7.9	–	–	0.4	10.2	–	51.1	–	–	–	–
(Chen et al. 2013)	Los Barrios (Spain)	2009	PM_2.5_	n.m.	–	6.0	–	–	0.3	8.5	–	44.7	–	–	–	–
(Fernández-Camacho et al. 2012)	Huelva (Spain)	IV 2008–XII 2009	PM_10_	32.7	5.3	2.3	9.6	600	0.3	3.7	45.3	47.4	6.2	0.7	31.9	14.4
(Fernández-Camacho et al. 2012)	Huelva (Spain)	IV 2008–XII 2009	PM_2.5_	19.3	3.4	1.6	4.0	200	0.2	2.3	31.2	37.3	5.1	0.6	19.7	10.8
(Fernández-Camacho et al. 2012)	Huelva (Spain)	IV 2008–XII 2009	PM_2.5–10_	13.4	1.9	0.7	5.6	400	0.1	1.4	14.1	10.1	1.1	0.1	12.2	3.6
(Moroni et al. 2012)	Terni (Italy)	XII 2008–XI 2009; winter	PM_1.3_	47	5.2	21	9.5	170	–	16	23	50	–	–	–	27
(Moroni et al. 2012)	Terni (Italy)	XII 2008–XI 2009; summer	PM_1.3–10_	23	2.1	16	16	940	–	11	28	47	–	–	–	9.9
(Moroni et al. 2012)	Terni (Italy)	XII 2008–XI 2009; winter	PM_1.3_	13	0.5	5.4	2.1	36	–	2.2	15	25	–	–	–	5.0
(Moroni et al. 2012)	Terni (Italy)	XII 2008–XI 2009; summer	PM_1.3–10_	16	0.2	5.5	3.0	115	–	2.4	5.6	25	–	–	–	2.4
(Contini et al. 2010)	Lecce (Italy)	I 2007–I 2008; average	PM_10_	26.3	1.6	2.2	8.0	306.0	–	3.0	12.9	24.8	<0.9	–	–	7.6
(Contini et al. 2010)	Lecce (Italy)	I 2007–I 2008; hot season (IV–IX)	PM_10_	27.2	2.1	2.5	9.8	365.8	–	3.6	13.0	19.5	<0.9	–	–	6.2
(Contini et al. 2010)	Lecce (Italy)	I 2007–I 2008; cold season (X–III)	PM_10_	25.2	1.0	1.8	5.8	236.1	–	2.3	12.8	31.3	<0.9	–	–	9.3
(Cuccia et al. 2011)	Carrara (Italy)	VII–IX 2007	PM_10_	34.57	6	4	13	583	–	3	15	33	–	–	–	5
(Dongarrà et al. 2007)	Palermo (Italy)	II–XI 2005	PM_10_	36	22	3.9	9.3	299	0.3	3.7	24	35	1.8	–	22	20
(Cuccia et al. 2013)	Genoa (Italy)	V 2009–V 2010	PM_10_	22	13	7	10	780	–	9	34	34	–	–	29	10
(Cuccia et al. 2013)	Genoa (Italy)	IV–V 2010	PM_2.5_	23	10	9	12	740	–	7	33	43	–	–	57	8
(Cuccia et al. 2013)	Genoa (Italy)	V 2009–V 2010	PM_10_	15	12	3	4	135	–	7	5	17	–	–	17	8
(Cuccia et al. 2013)	Genoa (Italy)	IV–V 2010	PM_2.5_	13	10	2	4	145	–	6	5	15	–	–	22	6
(Lettino et al. 2012)	Tito Scalo (Italy)	IV 2010	PM_2.5_	9	–	47	9	195	3.0	16	8	420	–	2	–	34
(Rogula-Kozłowska et al. 2013a)	Zabrze (Poland)	2007	PM_2.5_	22	0.2	1.7	16.1	160.8	0.3	0.8	6.5	72.5	1.5	1.2	6.4	24.8
(Rogula-Kozłowska et al. 2013a)	Katowice (Poland)	2007	PM_2.5_	31	0.5	1.8	8.8	157.0	0.4	0.4	8.2	90.3	2.1	2.3	4.2	35.1
(Rogula-Kozłowska et al. 2014)	Katowice (Poland)	2010	PM_2.5_	43	–	–	–	–	–	1.2	–	–	1.1	1.1	–	24.5
(Rogula-Kozłowska et al. 2014)	Gdańsk (Poland)	2010	PM_2.5_	24	–	–	–	–	–	1.4	–	–	0.7	0.9	–	22.3
(Majewski et al. 2011)	Warsaw (Poland)	2007–2008 average	PM_10_	27.4	–	–	–	–	–	3.54	–	–	0.14	0.96	–	–
(Zwoździak et al. 2013)	Wrocław (Poland)	XII 2009–X 2010; winter	PM_2.5_	55	–	4.3	23	215	–	4.0	40	227	4.9–	–	–	81
(Zwoździak et al. 2013)	Wrocław (Poland)	XII 2009–X 2010; summer	PM_2.5_	11	–	1.6	9	78	–	0.7	20	43	1.2	–	–	27
(Szoboszlai et al. 2012)	Debrecen (Hungary)	X 2008	PM_2.5–10_	13.7	–	–	11.1	655.4	–	–	34.6	16.9	–	–	–	6.8
(Szoboszlai et al. 2012)	Debrecen (Hungary)	V 2009	PM_2.5–10_	9.3	–	–	5.1	222.2	–	–	8.5	3.3	–	–	–	1.5
(Szoboszlai et al. 2012)	Debrecen (Hungary)	X 2008	PM_2.5_	11.4	–	–	7.9	353.8	–	–	15.5	38.4	–	–	–	30.2
(Szoboszlai et al. 2012)	Debrecen (Hungary)	V 2009	PM_2.5_	7.5	–	–	2.1	111.6	–	–	8.0	5.3	–	–	–	2.4
(Sánchez-Jiménez et al. 2012)	London (UK)	VIII 2006	PM_10_	25.5	1.49	–	0.352	5.04	–	0.803	1.56	7.73	0.524	0.061	–	2.44
(Sánchez-Jiménez et al. 2012)	Glasgow (UK)	VIII 2006	PM_10_	21.6	0.183	0.141	1.57	26.0	–	0.473	3.06	–	0.040	0.054	–	0.714
(Witt et al. 2010)	Oxford (UK)	XI 2007–III 2008	PM	n.m.	0.7	1.2	3.5	122	0.22	67.3	39.5	30	–	1.06	–	186
(Tecer et al. 2012)	Zonguldak (Turkey)	XII 2004–X 2005	PM_2.5–10_	24.9	–	3.7	12	352	–	2.9	60	26	–	–	–	7.3
(Tecer et al. 2012)	Zonguldak (Turkey)	XII 2004–X 2005	PM_2.5_	29.6	–	3.8	8	130	–	3.0	61	58	–	–	–	11.9
(Theodosi et al. 2010)	Istanbul (Turkey)	XI 2007–VI 2009	PM_10_	39.1	0.014	0.004	0.02	0.70	–	0.007	0.020	0.24	–	0.001	–	0.07
(Byrd et al. 2010)	County Cork (Ireland)	Summer 2005	PM_10_	14	–	4	4	203	bld	12	4	80	–	bld	bld	8
(Byrd et al. 2010)	County Cork (Ireland)	Spring 2006	PM_10_	14	–	–	–	–	–	–	–	–	–	–	bld	–
(Gu et al. 2011)	Augsburg (Germany)	Winter 2006/2007	PM_10_	31.77	–	9.4	15.7	1261	0.23	3.9	43.9	47.4	–	0.19	–	8.8
(Gianini et al. 2012)	Zurich (Switzerland)	VIII 2008–VII 2009	PM_10_	20.7	0.6	2.0	5.6	420	–	1.0	20.6	28.2	0.52	0.12	3.7	5.2
(Vercauteren et al. 2011)	Borgerhout (Belgium)	IX 2006–IX 2007	PM_10_	33.7	9.6	4.5	10.5	1040	–	4.6	24.6	72	17.5	–	–	27.0
Present study	Zabrze (Poland)	IX–XII 2009	PM_10_	36.35	–	4.61	62.61	260.79	–	0.26	19.43	109.67	2.5	1.22	5.71	36.2

*n.m.* no measurement, *bld* below limit of detection

^a^PM concentration in μg/m^3^

Usually, the PM investigations focus on the elemental composition of PM_2.5_ or PM_10_ (*D*_p_ ≤ 2.5 or 10 μm). While such works are quite numerous (not only in Europe, also in Asia and the Americas), the elemental composition of the submicron (PM_1_) and ultrafine (PM_0.1_) PM has not been investigated very intensely, neither has the distribution of the PM-bound element masses among the PM fractions (Sanderson et al. 2014). However, these few existing studies prove that most of the mass of the PM-bound toxic metals accumulates in the finest particles (e.g., Birmili et al. 2006; Arhami et al. 2009; Daher et al. 2014; Rogula-Kozłowska et al. 2013b, c; Table 1). In fact, the finer PM particles are, the more they are toxic. It combines with the ability of the inhaled finest particles to reach the pulmonary alveoli, where the absorption efficiency for elements is 60–80 % (Infante and Acosta 1990; Pope and Dockery 2006). Such particles make usually the main part of the PM mass in urbanized areas, also in Poland (Klejnowski et al. 2007, 2012). Therefore, the analysis of the elemental composition of the finest PM particles provides essential information on the PM toxicity (Almeida et al. 2007; Kumar et al. 2008; Daher et al. 2012, 2014).

In a heating season, in most (European) urban areas, the combustion of various fuels for heating keeps the PM concentrations high (Table 1). In Poland, especially in its southern part, in winter (January and February), episodes of smog and extremely high PM concentrations are frequent; they are due to the municipal emissions and specific weather conditions (Pastuszka et al. 2003, 2010; Juda-Rezler et al. 2011; Klejnowski et al. 2007, 2012; Lewandowska and Falkowska 2013; Rogula-Kozłowska et al. 2013b, 2014; Nidzgorska-Lencewicz and Czarnecka 2014). For the purposes of this study, PM was sampled in Zabrze, a city in southern Poland, in autumn and winter (September–December of 2009), when all the possible emission sources are active. The emissions from traffic, industry, and re-suspended dust are present in Zabrze during the whole year; the municipal emissions (fossil fuel combustion) grow significantly in late autumn and early winter, but in September–December the smog episodes and very high municipal emissions, capable of overshadowing the contributions of other sources to the PM elemental composition and concentrations, almost certainly do not occur. Such a selection of the sampling period favored the determinations of the mass distributions of the 18 PM-bound elements with respect to particle size and of the correlations between the PM-bound elements.

The aim of the work is to give some insight into the origin of particular PM fractions in an urban area. This is achieved by analyzing the modalities of the mass distributions of PM and 18 PM-bound elements (S, Cl, K, Ca, Cr, Mn, Fe, Ni, Cu, Zn, Ge, As, Br, Sr, Cd, Sb, Ba, and Pb) among 13 PM size fractions and application of cluster analysis (CA) to the first principal components (PC1) found for 13 fraction-related sets of concentrations of these elements using principal component analysis (PCA). The results are discussed and interpreted in terms of the source apportionment of PM emissions.

## Materials and methods

**Fig. 1:**
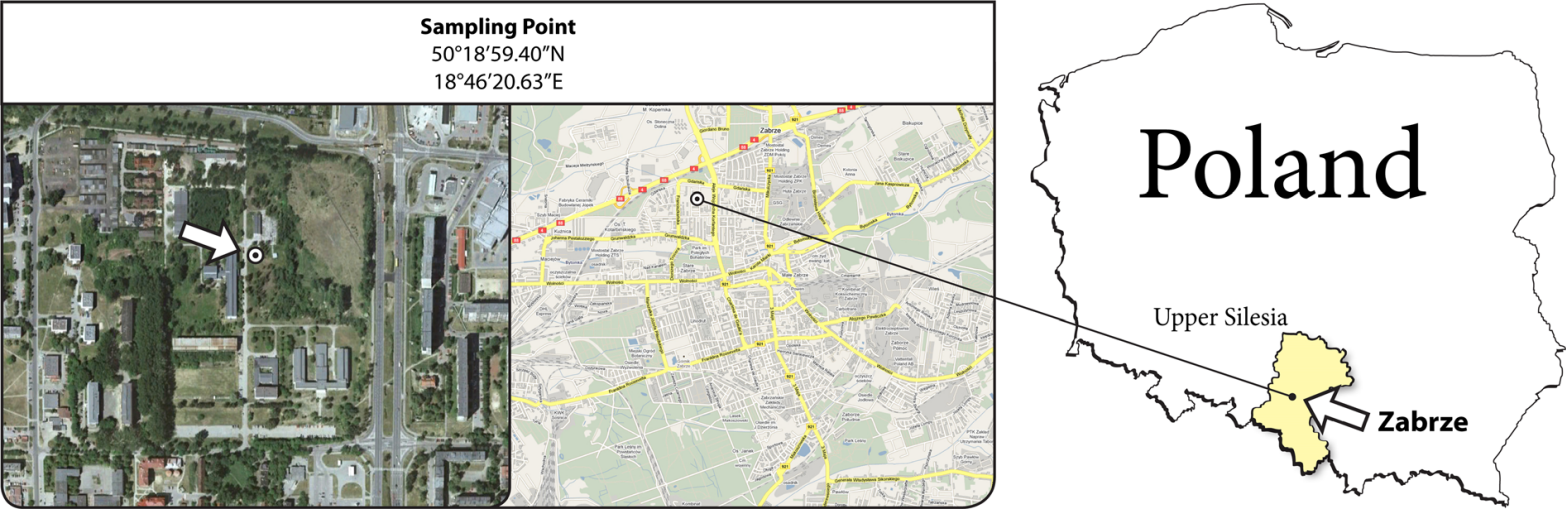
The sampling point location

PM was sampled in Zabrze (southern Poland, Silesia Province), between 5 September and 25 December of 2009, at an urban background site (EC 2008; Fig. 1). The sampling point neighborhood consisted of a trunk road (approx. 500 m to the north), blocks of flats and residential houses (to the east), Zabrze city center with residential and commercial housing (to the south and south-east), and blocks of flats and allotments (to the west). The surrounding houses were either connected to the district heating network or heated with local coal-, gas-, or electricity-powered boilers.

The city of Zabrze (80.5 km^2^, 2363 person/km^2^) is located in the Upper Silesia Agglomeration. In Zabrze, the degradation of the environment related to hard coal excavation and production of coal-derivatives started in the early nineteenth century (a cokery built in Zabrze in 1884 still works, some other, more ancient, were shut down in the late twentieth century). The problem of the environmental pollution in the area was considered serious late, in the 1950s (Pastuszka 1997), and even this did not stem the further environmental degradation, most effective during the 1960s and 1970s. In the 1970s, the dust deposition in Zabrze exceeded 2000 g/(m^2^ · year); in the 1980s, the lead deposition reached 3 g/(m^2^ · year) in the Zabrze surroundings (Rogula-Kozłowska et al. 2012; references therein). Although the industrial emissions have been seriously reduced during the last 30 years, the PM concentrations within the area are still one of the highest in the EU (Table 1), mainly because of dense and obsolete housing, where poor quality hard coal, scrap, and household garbage are burnt in inefficient ovens to warm flats.

The PM samples were collected with a 13-stage low-pressure cascade impactor (DLPI, Dekati). The impactor catches particles of the aerodynamic diameter (*D*_p_) from 0.03 to 40 μm at the flow rate of 1.8 m^3^/h (pump: Sogevac SV/25). It splits PM into 13 PM fractions: PM_0.03–0.06_, PM_0.06–0.108_, PM_0.108–0.17_, PM_0.17–0.26_, PM_0.26–0.4_, PM_0.4–0.65_, PM_0.65–1.0_, PM_1.0–1.6_, PM_1.6–2.5_, PM_2.5–4.4_, PM_4.4–6.8_, PM_6.8–10.0_, and PM_10.0–40_. PM_x-y_ denotes the PM fraction whose particles have their *D*_p_ in the interval [x,y]; *D*_p_ = 0.03 μm in PM_0.03–0.06_ and *D*_p_ = 40 μm in PM_10.0–40_ are the *D*_p_ limits for particles possible to sample (Hinds 1998). PM_x_ stands for PM_0.03-x_. A single (continuous) sample-taking lasted 8–10 days. Altogether, 12 sample-takings were done that covered about 90 % of the whole sampling period.

The masses of PM on the substrates were determined gravimetrically, according to the European standards (PN-EN 12341:2006a; PN-EN 12341:2006b). Before each weighing of the substrates, before and after their exposure, they were conditioned for 48 h in the weighing room (air temperature 20 ± 1 °C, relative air humidity 50 ± 5 %RH). A concentration of a PM fraction was calculated from its mass on a substrate and from the flow rate.

The PM samples (156 in total) were analyzed for their elemental content by means of energy dispersive X-ray fluorescence (EDXRF) on a PANalytical Epsilon 5. The apparatus was equipped with an X-ray tube with a side window (nitrogen-cooled, gadolinium anode, working range 25–100 kV, 150-μm beryllium window), a system of nine secondary targets (Al, Ti, Fe, Ge, Zr, Mo, Ag, Ce_2_O_3_, Al_2_O_3_), and a Ge(Li) detector (resolution 140 eV, energy range 0.7–100 keV, working surface 30 mm^2^, 8-μm beryllium window).

The measurements were conducted under vacuum. The analysis, including the run of the whole program of the changes of targets and X-ray tube settings (25 keV and 25 mA for Al, 40 keV and 15 mA for Ti, 40 keV and 15 mA for Fe, 75 keV and 8 mA for Ge, 100 keV and 6 mA for Zr, 100 keV and 6 mA for Al_2_O_3_) lasted 2400 s. The concentrations of particular analytes were derived by comparing the results with the calibration curves. The curves were determined by measuring thin-layer standards (Micromatter, Inc.) and corrected for possible matrix effects.

The NIST standard reference material (SRM2873) was measured weekly. The recovery of each element was between 84 % (As) and 116 % (Pb) of the certified value. Three blanks (aluminum substrates) were used to determine the detection limits for the procedure. Each blank underwent the entire EDXRF procedure devised for a regular sample 30 times; a detection limit for an element was the standard deviation from the 90 results obtained for this element. The detection limits were between 0.3 (Pb) and 11.6 (As) ng/cm^2^.

## Results and discussion

Table 2 shows the average (over the measuring period) ambient concentrations of 13 PM fractions and of the fraction-bound elements. The average PM_10_ concentration, 36.3 μg/m^3^, is comparable with or slightly higher than the PM_10_ concentrations in other European urban locations in winter (Table 1). In Zabrze and other Upper Silesian cities, the PM concentrations can be significantly higher at the turn of the year and in January–February (Pastuszka et al. 2010; Rogula-Kozłowska et al. 2014).

**Table 2:** The average concentrations of 13 PM fractions (μg/m^3^) and of the fraction-bound elements (ng/m^3^)

	0.03–0.06	0.06–0.108	0.108–0.17	0.17–0.26	0.26–0.4	0.4–0.65	0.65–1	1–1.6	1.6–2.5	2.5–4.4	4.4–6.8	6.8–10	>10
PM	0.15	0.38	0.76	2.21	3.72	8.38	8.37	5.34	2.35	1.98	1.41	1.30	1.67
S	4.04	4.70	5.66	11.83	22.22	56.07	69.43	60.64	27.67	13.14	9.45	7.86	11.54
Cl	4.95	4.62	10.00	24.24	48.08	135.09	140.85	103.9	41.24	36.51	34.76	23.78	25.62
K	0.88	1.89	2.67	6.99	10.93	21.58	20.22	18.17	11.21	12.69	11.55	13.43	18.78
Ca	0.09	0.04	0.08	0.09	0.11	0.32	0.83	4.87	12.72	26.56	30.91	40.66	55.79
Cr	0.28	0.29	0.28	0.31	0.33	0.42	0.47	0.52	0.47	0.43	0.39	0.42	0.61
Mn	5.57	5.27	5.35	5.01	4.92	4.61	5.23	5.97	5.4	5.13	4.96	5.19	6.12
Fe	<DL	<DL	<DL	<DL	0.40	7.48	10.29	25.85	36.07	61.21	55.72	63.77	86.29
Ni	0.02	0.01	0.02	0.02	0.02	0.05	0.04	0.02	0.01	0.01	0.02	0.02	0.03
Cu	1.13	1.11	1.12	1.32	1.46	2.29	2.17	2.02	1.73	1.87	1.67	1.54	1.57
Zn	0.84	1.21	1.83	4.98	8.9	25.61	25.52	19.96	7.65	4.92	3.83	4.42	6.16
Ge	0.002	<DL	0.01	0.06	0.23	0.59	0.55	0.31	0.07	0.02	<DL	<DL	0.004
As	0.09	0.07	0.11	0.09	0.23	0.63	0.46	0.33	0.11	0.16	0.05	0.17	0.04
Br	0.06	0.15	0.23	0.78	1.43	4.12	3.6	2.19	0.56	0.22	0.06	0.04	0.06
Sr	0.004	0.002	0.01	<DL	<DL	<DL	<DL	0.02	0.16	0.41	0.36	0.53	0.71
Cd	0.01	0.01	0.02	0.08	0.09	0.36	0.27	0.2	0.05	0.06	0.05	0.02	0.04
Sb	0.08	0.09	0.16	0.35	0.61	1.59	1.24	0.83	0.31	0.24	0.14	0.18	0.12
Ba	0.03	0.03	0.01	0.07	0.03	0.11	0.15	0.52	0.82	1.30	1.21	1.43	2.02
Pb	0.24	0.46	0.66	2.01	3.26	9.09	7.52	5.43	2.42	1.91	1.49	1.71	2.14
Σ_18_	18.32	19.95	28.22	58.23	103.25	270.01	288.84	251.75	148.67	166.79	156.62	165.17	217.64

*<DL* concentrations lower than the detection limits, *Σ*_*1*8_ the ambient concentration of the PM-bound total of the 18 elements averaged over the measuring period

**Fig. 2:**
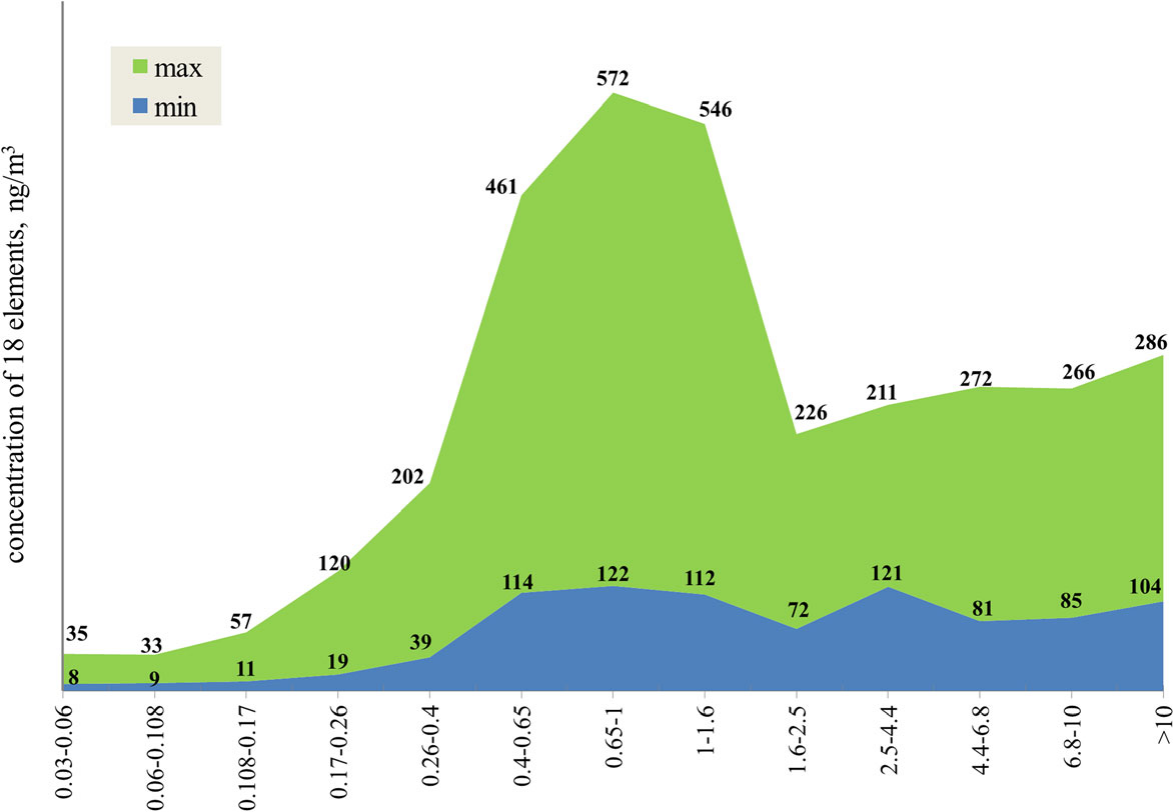
The ranges of the ambient concentrations of the total of the 18 element by PM fractions

Among all the fractions, PM_0.4–1_ had the highest concentrations, both those averaged over the measuring period and those from particular measurements. Like PM_0.4–1_ among the fractions, the total of the 18 PM_0.4–1_-bound elements had the highest mass concentration among the thirteen fraction-bound 18 element totals. Somewhat lower, but also high, was this concentration for PM_1–1.6_. The differences between minimum and maximum of the collective ambient concentrations of the 18 elements in the measuring period are the largest for PM_0.4–0.65_, PM_0.65–1_, and PM_1–1.6_, the sub-fractions of PM_0.4–1.6_, and the smallest for PM_0.03–0.06_, PM_0.06–0.108_, and PM_0.108–0.17_, the sub-fractions of PM_0.03–0.17_ (Fig. 2). For the latter, the finest, these concentrations are very low—the measuring period maximum for PM_0.06–0.108_ is 19 times lower than for PM_0.65–1_. Instead, the proportions of the measuring period maximum to minimum of these concentrations for all the sub-fractions of both PM_0.4–1.6_ and PM_0.03–0.17_ are close, about 4–5; they are about 3 for PM_4.4–6.8_, PM_6.8–10_, and PM_>10_, contained in PM_>4.4_. The measuring period averages of the 18 element total concentrations for the sub-fractions of PM_>4.4_ are significantly higher than those for the smallest particles (Table 2), but they differ less between the particular measurements. For example, the maximum to minimum proportions of the 18 element total concentrations for PM_1.6–2.5_ and PM_2.5–4.4_ are low, about 2 and 3; for PM_0.17–0.26_ and PM_0.26–0.4_ they are about 5 and 6, respectively (Fig. 2).

By weight, the shares of the 18 element totals in PM_1.6–40_ and in PM_0.03–0.108_ were the greatest among all the fractions. Their averages over the measuring period are 10 and 9 %, respectively. PM_0.4–1_ had the highest mass concentration in the air; however, the 18 elements made less than 3.5 % of the PM_0.4–1_ mass.

Non-metals S and Cl and crustal Fe, Ca, and K dominated in every of the 13 basic PM fractions. S and Cl accumulated in PM_2.5_. Fe, Ca, and, also crustal but occurring at concentrations lower than other crustal elements, Sr accumulated in coarse PM; for example, PM_2.5–40_-bound Fe was 77 % of the total PM-bound Fe mass. On the other hand, the K contents of both the coarse particles and PM_0.26–2.5_ were high (Table 2).

The elements in fine PM come mainly from combustion (car engines, power plants, waste incinerators, household ovens, etc.). Coarse dust contains metals from natural sources (Chow et al. 1995). Iron is the most ubiquitous component of the Earth crust, and its greater ambient concentrations are associated with the occurrence of coarse rather than fine PM, like those of silicone or aluminum that are also of the natural origin. However, power station fly dust can contain Al, Ca, and Fe in its mineral components as well (Chow et al. 1995).

The mass distribution between fine and coarse PM confirm the natural origin of the crustal Fe, Ca, and Sr in September–December 2009 in Zabrze (soil re-suspension). However, K, which is also crustal, could have come from two sources: soil re-suspension and fuel combustion (Table 2; Chow 1995; Querol et al. 2007; Duvall et al. 2008; Rogula-Kozłowska et al. 2013a, c; 2014).

The measuring period averages of the ambient concentrations of the remaining elements depend on a fraction. The highest, those of the PM-related Mn, Zn, and Pb, equal to 69, 116, and 38 ng/m^3^, respectively, are mainly due to Mn, Zn, and Pb contents in fine particles. The Zn concentrations in Zabrze are comparable with, or even lower, than those in other European urban locations. Instead, the Mn and Pb concentrations in Zabrze are among the European highest (Table 1). The PM-related Cr, As, and Cd concentrations were similarly high; their measuring period averages are 5.2, 2.5, and 1.3 ng/m^3^, respectively. The ambient concentrations of these elements, although relatively high, did not exceed their ambient permissible values (WHO 2000). However, the health hazard from them could have been greater than this estimated from their concentrations because most of their mass was in very fine particles. In general, metals were occurred mostly in the fine PM fractions. The average mass shares of particular PM_1_-bound metals in their PM-bound totals range between 45 % (Cr) and 78 % (Ge). PM_2.5_ contained yet more metals—between 65 % (Cr) and 99 % (Ge) of the total metal content of PM.

**Fig. 3:**
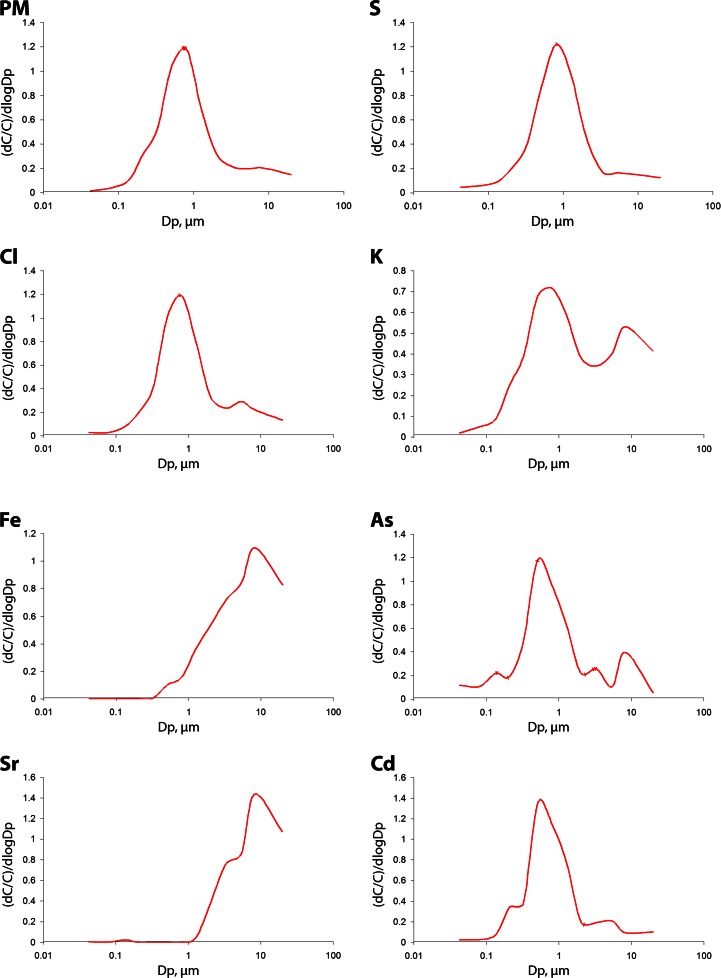
The mass size distributions of the selected PM-bound elements (*dC* fraction-bound element concentration, *C* average concentration of PM-bound element, *Dp* particle aerodynamic diameter)

The density functions of the mass size distributions of PM and seven selected elements are presented in Fig. 3. Table 3 presents all the particle *D*_p_ intervals in which the maxima of these functions occur, for all the discussed elements and PM.

**Table 3:** The particle size intervals in which the maxima of the mass size distributions of PM and PM-related elements occur

	Max 1	Max 2	Max 3	Max 4
PM	0.65–1	6.8–10		
S	0.65–1			
Cl	0.65–1	4.4–6.8		
K	0.65–1	6.8–10		
Ca	6.8–10			
Cr	1–1.6	6.8–10		
Mn	0.108–0.17	1–1.6	6.8–10	
Fe	6.8–10			
Ni	0.4–0.65	6.8–10		
Cu	0.65–1	6.8–10		
Zn	0.65–1	6.8–10		
Ge	0.65–1			
As	0.108–0.17	0.4–0.65	2.5–4.4	6.8–10
Br	0.4–0.65			
Sr	0.108–0.17	6.8–10		
Cd	0.4–0.65	4.4–6.8		
Sb	0.4–0.65	6.8–10		
Ba	0.06–0.108	0.17–0.26	6.8–10	
Pb	0.4–0.65	6.8–10		

The density function of PM mass distribution with respect to particle size at a given receptor has usually several maxima (modes; 2, 3, less often 4; Hinds 1998). Each maximum characterizes a population of particles (also called mode). Each population has its separate distribution (lognormal; Whitby 1978; Whitby and McMurry 1997). The populations partly overlap, and the set of those distributions is characteristic of the place in which it occurs.

The mass size distribution of PM in Zabrze for the heating period of 2009 is bimodal (Fig. 3). The first maximum of its density function occurs between 0.65 and 1 μm (accumulation mode; Fig. 3, Table 3), the second between 6.8 and 10 μm (coarse mode). It corroborates the results of earlier studies (Klejnowski et al. 2012) that suggest that two particle mass populations dominate the PM in Zabrze. They are fine primary PM originating from combustion, and coarse PM from re-suspended road and soil dust, erosion of various materials, etc. (Chow 1995; Hinds 1998; Karanasiou et al. 2011; Kumar et al. 2013; Pant and Harrison 2013).

The numbers of the maxima of the density functions of the considered elements and the intervals of these maxima occurrence differ.

Eight elements (Cl, K, Ni, Cu, Zn, Cd, Sb, Pb), like PM, have bimodal distributions, with one maximum within the interval of great *D*_p_ (most often between 6.8 and 10 μm; for Cl and Cd between 4.4 and 6.8 μm) and the second one within the small *D*_p_ (between 0.4 and 1 μm). Therefore, these PM-bound elements could have come from anthropogenic sources (fossil fuels and biomass combustion, accumulation mode), natural sources (mineral dust), and mechanical processes (brake system wear and road dust re-suspension; coarse mode) (Garg et al. 2000; Sternbeck et al. 2002; Adachi and Tainosho 2004; Slezakowa et al. 2007; Gaudry et al. 2008; Pant and Harrison 2013), having multiple origins. In general, Zn, Ni, Cu, Sb, Cd, and Pb occur in car exhaust (Chow 1995; Geller et al. 2006; Maricq 2007; Dias da Silva et al. 2008; Pant and Harrison 2013); some researchers consider Zn an indicator of the road dust re-suspension (tire wear) in urban areas (Amato et al. 2009, 2011).

Each, the bimodal Cr and the three-modal Mn mass size distributions, have two modes in *D*_p_ >1 μm. Probably, these elements came from fragments of brake linings and tires in re-suspended road dust (Kupiainen et al. 2005; Wahlin et al. 2006; Amato et al. 2009, 2011; Harrison et al. 2012; Pant and Harrison 2013). Mn, like As and Ba, might have come from the nucleation processes—the maxima of the density functions of their distributions occur within the interval of the smallest *D*_p_ (Table 3; Fig. 3). The regularly shaped particles of metal oxides, being the condensation centers for the finest PM, indicate the influence of high-temperature processes (e.g., steel and iron working; Moreno et al. 2004; Mogo et al. 2005) or of road traffic (Geller et al. 2006; Maricq 2007) on the formation of the PM particles.

The mass size distributions of S, Ge, and Br are unimodal; the maxima occur between 0.65 and 1.0 μm for S and Ge, and between 0.4 and 0.65 μm for Br. It means that the airborne S, Ge, and Br in Zabrze were anthropogenic.

The behavior of the ambient crustal element concentrations confirms the natural origin of greater parts of airborne Fe, Ca, and Sr (soil re-suspension). However, while the mass size distributions of Fe and Ca, typical crustal elements coming solely from soil matter, are unimodal that of Sr is bimodal (Fig. 3; Table 3). Each of the three density functions has one maximum between 6.8 and 10 μm; the Sr distribution has also the maximum between 0.108 and 0.17 μm. This bimodality of the Sr distribution indicates the existence and effects of an anthropogenic Sr source(s) affecting the measuring point in Zabrze.

The possible anthropogenic effect on the ambient concentrations of the PM-related elements can be assessed by computing enrichment factors (EF, Table 4). The enrichment factor EF_x_ for the element *x* is defined as:

1 $$ {\mathrm{EF}}_x=\frac{{\left({C}_x/{C}_{\mathrm{ref}}\right)}_{\mathrm{PM}}}{{\left({C}_x/{C}_{\mathrm{ref}}\right)}_{\mathrm{crust}}} $$

where *C*_*x*_ and *C*_ref_ are the concentrations of the element *x* and the reference element, and (*C*_*x*_/*C*_ref_)_PM_ and (*C*_*x*_/*C*_ref_)_crust_ are the proportions of these concentrations in PM and in the Earth crust, respectively. Ca was assumed as the reference element, i.e., EF_Ca_ = 1. The chemical composition of the upper continental crust was taken from (Wedepohl 1995). The closer EF_x_ is to 1, the weaker are the anthropogenic effects on the element x ambient concentrations.

**Table 4:**
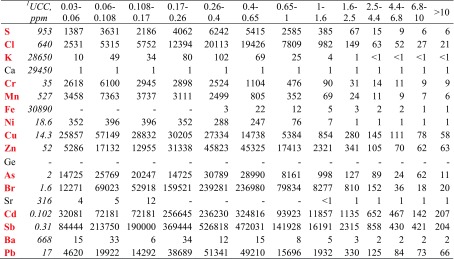
The enrichment factors (EF) for the elements in 13 PM fractions

^1^Element content (ppm) in the upper continental crust; data taken from (Wedepohl 1995); the symbols in red are the symbols of elements whose EFs for some fractions are high, indicating the anthropogenic effect on these element contents in PM

The EF analysis confirmed the origin of the PM-bound elements in Zabrze. The PM_1_-bound elements (except Fe, Sr, and Ba) have very high EFs, the coarse PM-bound ones—much lower; thus, it is certain that the anthropogenic emissions account for their contents in fine PM. The measurement period and the sampling point location suggest that combustion of fuels in household ovens and car engines were their sources. It is also true of Fe, which, despite its unimodal distribution (maximum within the interval of great *D*_p_) and low EFs for the fractions of particles with *D*_p_ > 1 μm, has higher EFs for the sub-fractions of PM_0.4–1_.

S, Cl, Cr, Mn, Cu, As, Br, and Pb in coarse PM could have come from re-suspended polluted soil or road dust. Low, not greater than 1, EFs for coarse PM-bound K, Ni, Sr, and Ba prove mineral matter (soil) to have been the main source of these elements in coarse PM.

Principal component analysis^1^ (PCA; Larose 2012) was applied separately to each of the 13 PM fraction-related sets of the 18 element concentrations, taking the 18 PM-bound element concentrations as the predictors. Only from 3 to 5 first principal components had their eigenvalues greater than 1 (Guttmann-Kaiser criterion, Kleinbaum et al. 1998; Larose 2012). Together, these PCs account for 90.1 % (PM_0.4–0.65_) to 99.7 % (PM_4.4–6.8_) of the fraction data set variance, the PC1s contribute from 42 to 69 % to it, and only one PC2, that for PM_0.06–0.108_, contributes more than 30 %. The remaining PC2s contribute no more than 17 to 23 %, depending on fraction, and no PC3, PC4, or PC5 contribute more than 15 %. The loadings for all the 13 PC1s are presented in Table 5.

**Table 5:** The loadings of 13 fraction-related PC1

	0.03–0.06	0.06–0.108	0.108–0.17	0.17–0.26	0.26–0.4	0.4–0.65	0.65–1	1–1.6	1.6–2.5	2.5–4.4	4.4–6.8	6.8–10	>10
S	*0.93*	*−0.94*	*0.93*	*−0.97*	*−0.85*	−0.67	−0.70	*−0.89*	*−0.88*	−0.51	*−0.76*	*−0.95*	*−0.90*
Cl	*0.95*	*−0.74*	*0.80*	*−0.75*	*−0.85*	−0.66	−0.66	*−0.82*	0.12	0.43	0.04	−0.29	0.41
K	*0.77*	*−0.97*	*0.96*	*−0.90*	*−0.94*	*−0.94*	*−0.91*	*−0.92*	*−0.96*	*−0.92*	*−0.94*	*−0.89*	*−0.97*
Ca	0.58	−0.54	−0.47	−0.33	−0.66	*−0.74*	*−0.75*	−0.67	*−0.79*	*−0.82*	*−0.96*	*−0.94*	*−0.99*
Cr	*0.84*	−0.46	0.35	−0.52	*−0.76*	*−0.76*	*−0.94*	*−0.80*	−0.10	−0.35	*−0.82*	*−0.82*	*−0.89*
Mn	0.62	0.04	−0.30	−0.14	0.05	−0.20	−0.62	−0.64	−0.54	−0.41	−0.30	−0.28	−0.45
Fe	–	–	–	–	0.52	−0.45	*−0.87*	*−0.89*	*−0.94*	*−0.94*	*−0.98*	*−0.96*	*−0.96*
Ni	*0.82*	−0.67	0.38	−0.68	*−0.77*	*−0.85*	*−0.96*	*−0.86*	0.14	0.16	−0.62	−0.66	*−0.70*
Cu	*0.86*	*−0.78*	0.64	*−0.91*	*−0.94*	*−0.95*	*−0.96*	*−0.93*	*−0.91*	*−0.92*	*−0.84*	*−0.90*	−0.62
Zn	*0.93*	*−0.98*	*0.96*	*−0.95*	*−0.96*	*−0.97*	*−0.95*	*−0.96*	*−0.98*	*−0.76*	*−0.94*	*−0.97*	*−0.96*
Ge	0.16	–	0.51	−0.37	*−0.87*	*−0.72*	−0.63	*−0.76*	*−0.75*	−0.36	–	–	0.12
As	0.67	0.69	0.46	−0.30	−0.51	0.07	0.46	0.14	−0.22	0.04	−0.52	0.36	−0.14
Br	*0.86*	*−1.00*	*0.98*	*−0.96*	*−0.99*	*−0.96*	*−0.93*	*−0.97*	*−0.81*	−0.49	−0.54	*−0.87*	*−0.86*
Sr	−0.13	−0.24	0.45	–	–	–	–	0.28	−0.24	−0.67	*−0.89*	*−0.95*	*−0.93*
Cd	−0.46	−0.46	−0.36	*−0.80*	−0.29	*−0.93*	*−0.82*	−0.24	0.66	*0.85*	−0.44	−0.21	−0.41
Sb	0.25	−0.46	*0.80*	−0.68	*−0.87*	*−0.95*	*−0.95*	*−0.98*	*−0.89*	−0.65	*−0.79*	*−0.91*	−0.11
Ba	0.26	−0.10	−0.35	−0.61	0.09	−0.58	*−0.87*	*−0.85*	*−0.97*	*−0.97*	*−0.96*	*−0.96*	*−0.82*
Pb	*0.80*	*−0.94*	*0.97*	*−0.99*	*−0.97*	*−0.95*	*−0.96*	*−0.96*	−0.63	0.27	*−0.80*	*−0.85*	*−0.78*
Eigenvalue	8.24	7.75	7.81	8.51	9.80	10.15	11.79	11.38	9.22	7.56	9.80	10.81	9.69
Variance, %	48	48	46	53	58	60	69	63	51	42	58	64	54

Loadings whose absolute values are greater than 0.7 are indicated in italics

**Fig. 4:**
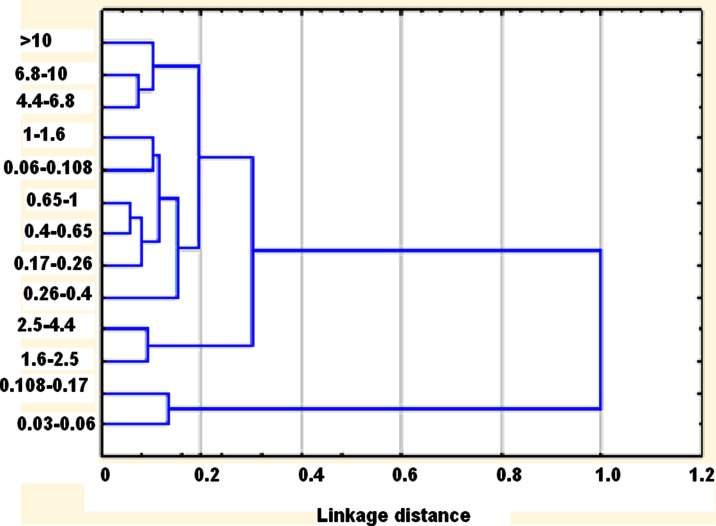
Dendrogram of cluster analysis (Ward’s method): the basic PM fractions grouped by similarity of their probable origin

For each of the basic 13 PM fraction, its PC1 represents its most effective source (Hopke et al. 1976; Thurston and Spengler 1985; Sówka et al. 2012). The fractions were grouped by applying cluster analysis (CA) to the 13 first principal components received from PCA (Fig. 4; Ward’s method, minimum variance criterion; Larose 2012; Kleinbaum et al. 1998). Such an application of CA allowed for grouping the basic PM fractions in regard to their most probable origin in four clearly distinguished clusters (Fig. 4; Hopke et al. 1976): (PM_0.03–0.06_, PM_0.108–0.17_), (PM_1.6–2.5_, PM_2.5–4.4_,), (PM_0.06–0.108_, PM_0.17–0.26_, PM_0.26–0.4_, PM_0.4–0.65_, PM_0.65–1_, PM_1–1.6_), and (PM_4.4–6.8_, PM_6.8–10_, PM_>10_). These clusters match roughly the four particle populations revealed by the mass size distributions of PM and PM-bound elements, i.e., two sub-populations of coarse particles, accumulation population, and nucleation population, related to different sources.

For the five sub-fractions of PM_0.4_, the concentrations of S, Cl, K, Cu, Zn, Br, and Pb are highly correlated with PC1s, negatively for PM_0.17–0.26_, PM_0.26–0.4,_ and PM_0.06–0.108_, positively for PM_0.108–0.17_ and PM_0.03–0.06_ (except for Cu in PM_0.108–0.17_). The concentrations of PM_0.26–0.4_- and PM_0.03–0.06_-bound Cr and Ni, PM_0.26–0.4_-bound Ge, and PM_0.17–0.26_-bound Cd are also highly correlated with PC1s. The high correlation between S and PC1 in such fine particles may be due to the secondary aerosol content (Seinfeld and Pandis 1998), but the modality of the PM-bound S concentration distribution does not confirm this. The remaining PM_0.4_-bound elements correlated with PC1s originated mainly from car exhaust (Chow 1995; Birmili et al. 2006; Geller et al. 2006; Maricq 2007; Daher et al. 2014). Probably, they occurred as airborne metal oxides (condensation centers) or metal salts (presence of S, Cl, Br).

Two sub-fractions of PM_0.4_, PM_0.03–0.06_, and PM_0.108–0.17_ make one cluster. Their probable common main source might have been car exhaust. However, the three remaining PM_0.4_ sub-fractions are in one cluster with PM_0.4–0.65_, PM_0.65–1_, PM_1–1.6_, summing to PM_0.4–1.6_ (Fig. 4). The concentrations of the majority of the examined elements are highly correlated with PC1s for these three sub-fractions of PM_0.4–1.6_, like those for the sub-fractions of PM_0.4_ that are associated with combustion processes. CA suggests these processes to be the same as for PM_0.06–0.108_, PM_0.17–0.26_, and PM_0.26–0.4_, but different from the source of PM_0.03–0.06_ and PM_0.108–0.17_.

However, the PC1s for the sub-fractions of PM_0.4–1_, besides being negatively correlated with the concentrations of K, Cr, Ni, Cu, Zn, Br, Cd, Sb, and Pb, are also correlated with the concentrations of Ca. Because Ca has been shown to be of the natural origin (soil) in each fraction, the PM natural sources are supposed to have affected PM_0.4–1_.

Undoubtedly, most elements in the fine PM came from combustion. On the other hand, mineral matter and, probably, road dust were the sources of the majority of the elements in the coarse PM. For the sub-fractions of PM_1.6–40_, the PC1s (42 to 64 % of variance) are correlated with the concentrations of Ca, Fe, Sr, and Ba. Moreover, these PC1s are also correlated with the concentrations of S, K, Cr, Cu, Zn, Br, Sb, and Pb. Probably, the three coarsest PM fractions (*D*_p_ ≥ 4.4 μm), whose PC1s are correlated with Br and S, and with the metals Cr and Sb, were in part composed of re-suspended road dust (Amato et al. 2009; Karanasiou et al. 2011; Harrison et al. 2012). These fractions are in one cluster; they had a common main source (Fig. 4).

The two sub-fractions of PM_1.6–4.4_ make also one cluster. They differ from the coarser fractions in the lack of the correlations between their PC1s and Cr, Mn, Sr, and Pb, and for PM_2.5–4.4_ also between PC1 and S and Cl. These two fractions may have come from re-suspended soil polluted with metals.

However, the variety of PM sources in Zabrze and, in general, unknown elemental composition of the PM they release do not allow to exclude the effects of fuel combustion on the elemental composition of coarse PM (e.g., metal compounds adsorbed on soot particles); similarly, the effects of mechanical processes, such as wear of brake linings, tires, and road surface may have added some amounts of metals to ultrafine and fine PM (Sanderson et al. 2014).

## Summary

In the heating season of 2009, in an urban locality in Zabrze (southern Poland), PM-bound S, Cl, Fe, Ca, and K had the highest ambient concentrations among all the examined PM-bound elements. The concentrations of Mn, Zn, and Pb were the second highest. Most of the elements (except typically crustal) concentrated their masses in particles with *D*_p_ ≤ 1 (PM_1_). Majority of toxic metals had their greater PM mass shares in the finest PM; their toxicity combining with the physicochemical properties of the finest PM caused health hazard. The concentrations of the most toxic Pb, Cr, As, and Cd did not exceed the permissible values (WHO 2000), but, compared to the concentrations in other European cities, their ambient concentrations in Zabrze were high.

Determination of the particle size resolved elemental composition of PM, supporting the PM source apportionment, allowed to identify the hypothetic sources of PM in the particular particle size ranges. The identification was based on the analyses of the modality of mass size distributions of the PM-bound elements, principal component analysis (PCA), and cluster analysis (CA).

The PM mass size distribution in Zabrze for September–December 2009 is bimodal, it has the coarse and the accumulation modes. The mass size distributions of the PM-bound elements have from two to four modes, one and/or two coarse modes, and/or an accumulation mode, and/or a nucleation mode, depending on an element. The two coarse populations were probably the soil (mineral) particles and the re-suspended particles of the polluted soil or road dust (from the brake linings, tires, road surface, cars, etc.). The typical crustal elements (Ca, Fe, Sr, and Ba) characterized the particles of one coarse population, the same crustal elements and S, K, Cr, Cu, Zn, Br, Sb, and Pb were in the particles of the second. These two populations contained the particles with *D*_p_ > 1.6 μm. The particles with *D*_p_ between 0.108 and 1.6 μm, in accumulation mode, contained the elements S, Cl, K, Cu, Zn, Ge, Br, Cd, Sb, and Pb that are characteristic of fuel combustion in stationary furnaces (coal, biomass, and waste burned in obsolete household ovens; hard and brown coal-fired heating boilers in residential areas). The particles with *D*_p_ ≤ 0.108 μm in the nucleation mode could have also come from road traffic (car exhaust particles).

The source apportionment accomplished by using principal component (PCA) and cluster (CA) analyses was in agreement with that done by analyzing the modality of mass size distributions of the PM-bound elements.

There are four clear-cut groups of the PM fractions identified by applying CA to the 13 PC1s received from PCA: (PM_1.6–2.5_, PM_2.5–4.4_,), (PM_0.03–0.06_, PM_0.108–0.17_), (PM_0.06–0.108_, PM_0.17–0.26_, PM_0.26–0.4_, PM_0.4–0.65_, PM_0.65–1_, PM_1–1.6_), and (PM_4.4–6.8_, PM_6.8–10_, PM_>10_). These clusters match roughly the four particle populations revealed earlier by analyzing the mass size distributions of PM and PM-bound elements (two coarse particle populations, accumulation and nucleation populations related to different sources). Probably, road traffic (exhaust gases) was the basic source of PM_0.03–0.06_ and PM_0.108–0.17_ in the area; PM_0.06–0.108_, PM_0.17–0.26_, PM_0.26–0.4_, PM_0.4–0.65_, PM_0.65–1_, and PM_1–1.6_ came also from combustion but their main source was different from that of PM_0.03–0.06_ and PM_0.108–0.17_. Some of these fractions may have come partly from re-suspension of soil particles, like PM_2.5–4.4_, or of road dust, like PM_>4.4_.

## Footnotes

1 Calculated with Statistica (Stat Soft) and customized Eco Data Miner (Czechowski 2009).
